# Self-referential processing as the biological switch between classical and quantum functioning of the brain

**DOI:** 10.3389/fnhum.2026.1783138

**Published:** 2026-04-29

**Authors:** Josh Roeloffs, Jack A. Tuszynski

**Affiliations:** 1 Independent Researcher, Los Angeles, CA, United States; 2 Department of Mechanical and Aerospace Engineering (DIMEAS), Politecnico di Torino, Turin, Italy; 3 Department of Physics, University of Alberta, Edmonton, AB, Canada; 4 Allen Discovery Center, Tufts University, Medford, MA, United States

**Keywords:** anesthesia, brain dynamics, DMN, flow states, microtubules, quantum coherence

## Abstract

Dual-process theory of the brain distinguishes fast, parallel, late-commitment cognition (System 1) from slow, sequential, early-commitment cognition (System 2), yet lacks a mechanistic explanation for how these modes operate or how the brain switches between them. Quantum cognition research demonstrates that human decision-making follows quantum probability models under low confidence and classical Markov models under high confidence, suggesting a hybrid architecture where decoherence drives transitions between processing modes. We propose that self-referential evaluative monitoring functions as the biological switch that regulates the transition between System 1 and System 2 by modulating microtubule-mediated quantum coherence through electromagnetic boundary conditions. The causal chain proceeds from locus coeruleus-norepinephrine regulation of default mode network (DMN) activity, through electromagnetic field patterns generated by self-evaluation, to calcium-mediated modulation of the microtubule electrostatic environment. Recent experimental evidence indicates that microtubules exhibit quantum exciton energy migration comparable to photosynthetic complexes, with cooperative robustness increasing with system size. Because quantum coherence enables parallel exploration while classical processing offers stability, evolution would have selected for mechanisms that balance these demands. Anesthetics bind promiscuously yet selectively abolish consciousness, which is consistent with disruption of energy-threshold-dependent coherent processes in microtubules. The framework proposed in this paper leads to testable predictions relating DMN activity, flow states, insight, and confidence to shifts along the quantum-classical processing spectrum.

## 1 Introduction

### 1.1 The mechanism question in dual-process theory

Dual-process theory of the brain distinguishes fast, parallel processing (System 1) from slow, sequential processing (System 2), and this framework has become foundational to cognitive science (Kahneman and Frederick, 2005; Evans, 2011; Kahneman, 2011). Kahneman and Frederick (2005) describe System 1 and System 2 as “collections of cognitive processes that can be distinguished by their speed, their controllability, and the contents on which they operate,” where System 1 rapidly generates intuitive answers to judgment problems as they arise, while System 2 monitors the quality of the options generated, and it may either endorse, correct or override them. Evans (2011) connects System 2 processing to working memory and controlled attention, noting that many cognitive researchers link Type 2 processing to a central working memory resource (also known as controlled attention) which explains its slow, sequential and limited capacity operation. This characterization describes the correlates of dual-process cognition. However, there is still a need for a mechanistic explanation of how these processes work at an organ, cell and subcellular level. In particular, there is no clear explanation of what enables the brain to perform fast, parallel processing that appears to exceed sequential computational capacity. Furthermore, no mechanism has been put forward to explain how the brain can switch between these processing modes.

Penrose (1989, 1994) argued on firm conceptual grounds that human mind exhibits features such as mathematical understanding that cannot be replicated by any algorithmic process as he drew on Gödel’s incompleteness theorems to suggest that cognition must involve non-algorithmic physical processes. This has led him to speculate that human mind must employ quantum processes in at least some of its cognitive functions. Penrose’s collaboration with Hameroff has led to the development of a quantum physics-based model of quantum computation within neuronal microtubules (Hameroff and Penrose, 2014). The existence of quantum states in microtubules has been recently demonstrated in a series of very precise measurements (Kalra et al., 2023), which supports one of the two pillars of the Orch OR theory. The other pillar, namely gravitational self-collapse of the wavefunction, however, has been cast in doubt by lack of experimental validation (Donadi et al., 2021). As this paper will propose, the transition between quantum and classical processing in the brain may not require new physics or gravitational self-collapse, as the interaction between the two cognitive systems described above may itself regulate the timing and degree of decoherence through known electromagnetic and biochemical mechanisms. Recently, Busemeyer et al. (2019, 2020) demonstrated that human decision-making follows quantum probability models under certain conditions, which is consistent with these ideas. Their research strongly indicates that the quantum model produces more accurate predictions for low levels of confidence, while the Markov model with drift rate variability generates more accurate predictions for high levels of confidence. Together these results suggest that neither process alone, quantum or Markov, is sufficient to account for all conditions and all participants (Busemeyer et al., 2019).

Because neither model alone accounts for all conditions, these results may point toward the selection of a hybrid architecture where the brain employs different computational modes depending on the cognitive state, as the next section will explore. Busemeyer et al. (2019) model the transition between modes with an open system quantum formalism starting in a coherent regime (represented by a density matrix with off-diagonal terms) that decoheres into a classical Markov regime (represented by a density matrix with no off-diagonal terms). In their model, this transition occurs through interactions with a noisy mental environment, and Busemeyer et al. (2020) found that the type of cognitive operation affects this transition, as a binary decision for the first response may be more effective for producing what they describe as a “collapse,” analogous to quantum measurement, as compared to making a probabilistic judgment. Thermal noise in biological systems has been commonly used as an argument against the plausibility of quantum processes in the brain (Tegmark, 2000). However, if the noisy environment in Busemeyer et al. (2019) formalism represents a functional mechanism that drives transitions between processing modes, then thermal noise may be part of the switching process itself, and Section 2 will examine evidence that biological systems can harness decoherence in this way. The physical literature on the interaction between classical and quantum degrees of freedom is well developed, including foundational work on decoherence and einselection (Zurek, 2003), open quantum system dynamics (Breuer and Petruccione, 2002), and specific models of coupled classical-quantum systems (Bonilla and Guinea, 1992), which provides a rigorous foundation for future model development within this framework.

It is important to distinguish between quantum cognition as a mathematical modeling framework and quantum biology as a claim about physical mechanisms. The quantum probability models developed by Busemeyer et al. (2019, 2020) and Kvam and Pleskac (2017) demonstrate that quantum formalisms can describe decision-making patterns more accurately than classical models under certain conditions, but this mathematical success does not by itself require that physical quantum processes are occurring in neural tissue. The present paper proposes that this correspondence reflects an underlying physical substrate, and the sections that follow present the evidence supporting this proposal, and the energy allocation and electromagnetic boundary-condition mechanisms proposed here generate testable predictions that are independent of this interpretive commitment.

This paper proposes a candidate mechanistic framework that integrates findings from neuroscience, cognitive science, and quantum biology to account for transitions between parallel and sequential modes of cognition. The framework, without introducing new biological components, synthesizes existing empirical research into a unified control architecture that links self-referential evaluative processing, electromagnetic boundary conditions, and microtubule-scale quantum state dynamics. The aim is to articulate a physically plausible mechanism by which known neural and molecular processes could regulate the timing and degree of state commitment in cognitive processing. The sections that follow therefore review relevant empirical constraints and then assemble them into a coherent mechanistic proposal.

### 1.2 Evolutionary rationale for hybrid architecture

The finding that confidence level predicts which model performs better carries significant implications, as low confidence states favor quantum models while high confidence states favor classical Markov models (Kvam and Pleskac, 2017; Busemeyer et al., 2019, 2020). Kvam and Pleskac (2017) formalize this difference by describing how classical models represent beliefs about the value of a cue or criterion as binary values, either present [1] or absent [0]. This justifies an assumption that cognitive representations are characterized by discrete states, suggesting that only one set of beliefs or preferences is present at any instant of time. By contrast, quantum representations allow beliefs to exist in continuous superposition, which captures what Kvam and Pleskac describe as the “fuzzy impression” that is characteristic of uncertain beliefs before definite judgment forces collapse.

This difference has computational consequences, since Kvam and Pleskac (2017) conclude that a greater precision in representations must be balanced by greater storage and processing demands. They also observe that expertise development involves a characteristic transition during the acquisition of a specific expertise, which aligns with Kahneman and Frederick's (2005) observation of a transition between “effortful” and “effortless” action.

If cognition involves both quantum and classical processing, evolution may have faced a resource allocation problem, as quantum coherence offers computational advantages for exploring large possibility spaces but maintaining coherence requires continuous metabolic energy above a threshold of decoherence, while classical processing offers stability and communicable certainty but is computationally bounded. Vervaeke and Ferraro (2013) describe this trade-off as arising from competing evolutionary pressures with constraints on efficiency putting selective pressure on processing, in parallel with constraints on resiliency that enable new possibilities of processing. Considering this, evolutionary pressures may have favored organisms capable of dynamically balancing these demands.

The computational benefits of quantum-enhanced processing may be substantial, as Vervaeke and Ferraro (2013) illustrate the limits of classical search by noting that the number of alternative pathways to checkmate is 30^60^, which is approximately 4×10^88^. This number exceeds the number of atoms in the universe and yet it took a massive supercomputer, IBMs’ Deep Blue to finally beat a human (G. Kasparov) at chess in 1997. Kurian (2025) estimates that classical Hodgkin-Huxley action potentials process approximately 10^3^ operations per second in a neuron, while superradiant protein fibers in eukaryotic cells may reach approximately 10^13^ operations per second, i.e., ten billion times more. Kurian further suggests that biological quantum systems may operate within two orders of magnitude of the Margolus–Levitin bound, which represents the physical limit for computation. Patwa et al. (2024) support these estimates by concluding that classical energy demands could not account for the sub-neuronal information processing capacity of the brain, given its extremely low input power of only ~20 W. Cognitive abilities such as pattern recognition, insight, and expert intuition may depend on computational regimes that classical parallelism alone cannot support within biological constraints of space, time, energy and thermal stability.

Efficiency is not the only selection pressure, because hybrid processing would enable strategic advantages in uncertain and competitive environments. Quantum randomness provides a source of ontic uncertainty that classical deterministic mechanisms cannot replicate, and the ability to modulate between ordered and exploratory modes would itself be advantageous, which means evolutionary pressures may have favored mechanisms capable of shifting the brain along this spectrum. Carhart-Harris et al. (2014) provide empirical evidence consistent with this selection pressure, as they describe an inverted-U relationship between entropy and cognition where excessive order leads to rigidity and excessive entropy leads to disorder. Furthermore, they noted that normal waking consciousness positions brain dynamics close to criticality but at a slightly sub-critical point, which is consistent with an evolved system optimized to balance flexibility during exploration with order when decisive action is required. This raises the possibility that a biologically selected process controls the transition between these modes.

### 1.3 Mechanistic requirements for a biological switch

Dual-process theory successfully characterizes System 1 and System 2 at the cognitive level, but it does not explain how these modes are implemented or how transitions between them occur. This gap extends into neuroscience, as Plankar et al. (2013) reviewed evidence from systems neuroscience and biophysics that the rapid changes in functional neuronal connectivity necessary for cognition cannot be explained exclusively by synaptic mechanisms, and that coherent dynamics operating across multiple levels of neuronal organization may be required, although the mechanistic relationship between these levels has not been established.

Empirical work in quantum cognition shows that transitions between processing modes can be modeled as interaction with a noisy mental environment that produces decoherence in the mathematical formalism (Busemeyer et al., 2019), but without specifying the biological source of this noise or the means by which it is regulated. If the brain operates as a quantum-classical hybrid system, several mechanistic conditions must be satisfied. First, quantum coherence must be maintainable in biological tissue at physiological temperatures for sufficiently long periods of time. Second, there must exist a controllable mechanism that regulates transitions between coherence-dominated and decoherence-dominated processing regimes. Third, this mechanism must interface with metabolic energy allocation, since coherence maintenance and collapse-driven computation impose different energetic demands.

In this respect, Patwa et al. (2024) proposed that extended protein architectures within neuronal axons in the brain, may form a highly interconnected, ultrafast quantum-optical network. Operating at a quantum level of electromagnetic waves, this mechanism would be orders of magnitude faster than chemical (ionic) Hodgkin–Huxley-type transport which is currently used as a standard paradigm in neuroscience. The use of optical channels of neuronal communication was extensively modeled and deemed highly feasible from the physics point of view (Kumar et al., 2016) and found of potential use in learning processes in addition to speed (Zarkeshian et al., 2022).

The present paper proposes that self-referential evaluative processing, which operates through the Default Mode Network, fulfills the role of a hybrid quantum-classical mode of operation. By modulating the brain’s internal electromagnetic and ionic environment, self-evaluative processing would set the boundary conditions for microtubule quantum coherence dynamics and regulate the transition between quantum-enhanced and classical processing modes.

Because the complete pathway proposed here has not been tested as an integrated mechanism, the sections that follow assemble convergent evidence at three levels of empirical support which include direct experimental findings (e.g., Carhart-Harris et al., 2014; Cao et al., 2020; Kalra et al., 2023; Babcock et al., 2024; Khan et al., 2024; Patwa et al., 2024), physically motivated connections between these findings (e.g., Cavaglià et al., 2023; Pinotsis et al., 2023; Wiest, 2025), and the paper’s own hypothesis linking them into a single switching mechanism. Throughout the manuscript, indicative phrasing is reserved for empirically demonstrated findings and conditional phrasing is used for the framework’s proposals.

## 2 Quantum processes at the cellular level

Tegmark (2000) argued that quantum processes could not take place in the human brain, as the argument held that thermal fluctuations at physiological temperatures would destroy quantum coherence far too quickly to be functionally relevant. These criticisms have been challenged by subsequent analyses that identified fundamental misinterpretations (Hagan et al., 2002), and more broadly, the objection applied the constraints of closed equilibrium systems to biological tissue that operates as an open, energy-pumped system with fundamentally different dynamics.

The question of whether biology can use quantum mechanics has already been answered in other domains as quantum coherence in photosynthetic light-harvesting complexes at physiological temperatures has been well documented (Engel et al., 2007; Cao et al., 2020). Avian magnetoreception operates through a radical pair mechanism that depends on quantum effects for navigation (Hore and Mouritsen, 2016), and enzyme catalysis involves quantum tunneling in biological reactions (Klinman and Kohen, 2013). Quantum mechanics already underlies all of chemistry, including the molecular interactions that constitute biological function, and what the photosynthetic evidence demonstrated is that biological systems harness the interplay between coherence and decoherence as a functional mechanism, as Cao et al. (2020) found that Nature specifically exploits dissipation, and the decoherence it produces, to engineer energy transport to specific destination sites.

More recently, Nishiyama et al. (2024b) extended this line of reasoning and identified several additional problems with Tegmark’s calculations, which led them to revise the decoherence time estimates. Nishiyama et al. (2024a) found that super-radiant emission occurs at approximately 0.2 picoseconds, while thermal effects operate at approximately 10 picoseconds, i.e., two orders of magnitude more slowly. Since the time scales of superradiant emission of light are much shorter than those of thermal effects (~10 ps), super-radiance is achieved without thermal loss via a microtubule. The speed of the superradiant emissions, therefore, would allow for bursts of quantum computation to be faster than the noise that causes decoherence, which would enable these quantum processes to complete before decoherence would destroy them.

Most importantly, the afore-mentioned recent experiments by Kalra et al. (2023) showed the existence of quantum states in microtubules maintained at room temperature on the time scale of up to 5 ns.

Considering this, the landscape of relevant questions shifts from examining whether coherence can passively survive thermal fluctuations to whether the energy supply exceeds the thresholds necessary to maintain coherence as biological systems are pumped with metabolic energy in ways that standard quantum mechanics, which focuses almost exclusively on equilibrium systems, does not address. From this perspective, the brain operates more analogously to a laser than to an inert biological material in a test tube at room temperature, as coherent output requires continuous pumping above a threshold, and below that threshold the same physical substrate produces incoherent output. Therefore, the type of theoretical framework that should be considered is more in line with the work of Froehlich on biological coherence (Fröhlich, 1977).

### 2.1 Experimental evidence for microtubule quantum coherence

Kalra et al. (2023) conducted experiments that measured electronic energy migration within microtubules. Using tryptophan autofluorescence and an external fluorescence quencher (AMCA) to track energy migration, they found a diffusion length of the resulting quantum states in microtubules to be 6.64 ± 0.1 nm, which roughly corresponds to the size of a tubulin dimer. This is very efficient for structural proteins and the associated photoexcitation diffusion length in microtubules is substantially higher than that predicted by conventional Förster theory being comparable to that reported in some photosynthetic complexes. It is worth noting that the lattice-type structure of microtubules that incorporates periodic arrays of aromatic amino acids is qualitatively similar to the structure of molecular aggregates and light-harvesting complexes such as FMO complexes in chlorophyll.

Babcock et al. (2024) provided additional confirmation of collective quantum effects at physiological conditions, showing an enhancement of the fluorescence quantum yield from 12.4% for isolated tryptophan to between 15.7% and 19.5% for microtubules concluding that these networks exhibit collective and cooperative quantum effects that support robust quantum states in protein aggregates, such as microtubules under thermal equilibrium conditions. Moreover, Patwa et al. (2024) analyzed tryptophan chromophore networks across multiple neuroprotein structures including microtubules, actin filaments, and amyloid fibrils, and they found that all three structures exhibit highly superradiant states with quantum yields that remain robust to static disorder even at five times room-temperature energy levels, which demonstrates that quantum coherent effects are a general feature of neuroprotein architectures whose observable consequences increase with system size.

The standard assumption in quantum mechanics is that larger quantum systems decohere faster, as more components mean more opportunities for environmental interference to destroy quantum states. Babcock et al. (2024) and Patwa et al. (2024) found the opposite, as both demonstrate that the robustness of these systems to disorder increases with system size. This cooperative robustness means quantum effects actively strengthen as networks grow larger, which inverts the decoherence concern, as biological scale may actually help maintain coherence.

Given that quantum coherence has been shown to serve functional roles in photosynthetic energy transfer, avian navigation, and enzyme catalysis, and that microtubule tryptophan networks exhibit quantum properties comparable to photosynthetic complexes with cooperative robustness that increases with system size, the question of whether the brain also exploits these mechanisms becomes one of scope and specificity. If the brain does use quantum coherence for computation, then a mechanism for modulating this system based on cognitive demands would be expected. Additional evidence for the plausibility of the brain utilizing these quantum processes will be explored in Sections 4 and 5.

## 3 The proposed mechanism

The central proposal of this paper is that self-referential evaluative processing, operating through specific Default Mode Network (DMN) subsystems, generates electromagnetic field patterns that modulate quantum coherence in microtubules via membrane dipole dynamics and calcium signaling (Cavaglià et al., 2023; Pinotsis et al., 2023; Wiest, 2025). This would constitute a candidate biological switch between System 1 (quantum-enhanced parallel processing) and System 2 (classical sequential processing).

The most important variable in this framework is not DMN activity in a broad sense, but more specifically, it is the intensity of metacognitive self-evaluation. Menon (2023) describes how the DMN generates the “epistemic self” through distinct brain states and frames of thought central to the construction of the internal narrative, which combines episodic memory, language, and semantic memory processes in order to generate an ongoing internal narrative and subjective continuity of internal mental thoughts. Carhart-Harris et al. (2014) proposed that this self-organized DMN activity maintains the brain in a slightly sub-critical state through alpha oscillations and hierarchical control, and that disruption of this activity moves the system toward criticality with increased entropy and cognitive flexibility, as their finding that alpha power decreases in the PCC explained 66% of the variance in ego dissolution experiences under psilocybin points to a direct relationship between the intensity of DMN self-referential activity and the brain’s position on the order-entropy spectrum. Carhart-Harris and Friston (2019) characterize this self-evaluative capacity in terms of hierarchical predictive processing, where high-level priors encoded in the DMN constrain lower-level perception and cognition. Their REBUS model proposes that these high-level beliefs, which include beliefs about oneself and one’s relationship to the world, normally exert strong top-down influence that narrows the repertoire of possible cognitive states. This paper extends this as it proposes that when the self-evaluative processing is high, the electromagnetic environment within neurons would become noisier, which would act as the boundary conditions that accelerates decoherence in microtubule quantum states and pushes the system toward classical processing (Kalra et al., 2023; Babcock et al., 2024). Here, noise refers to increased environmental interaction and recording capacity at the molecular scale. When self-evaluative processing decreases, the internal electromagnetic environment becomes relatively quieter, which would allow quantum coherence to persist for longer periods of time and enable the parallel processing associated with System 1-like cognitive models (Busemeyer et al., 2019, 2020).

### 3.1 The complete causal chain

The proposed mechanism operates through a sequence of steps, and taken together, these steps form a continuous pathway from neural network activity down to quantum dynamics at the molecular level.

#### 3.1.1 Step 1. The LC-NE system regulates self-evaluative DMN activity

Van der Linden et al. (2021) identify the locus coeruleus-norepinephrine (LC-NE) system as central to regulating arousal and task engagement. This small nucleus in the brainstem, which is responsible for most of the brain’s norepinephrine release, appears to regulate “decisions on task engagement vs. disengagement” through different modes of norepinephrine release.

The LC-NE system operates in multiple modes that correspond to different cognitive configurations. In what they term the exploitation mode, the system promotes high task engagement and optimal engagement and performance (van der Linden et al., 2021). This mode emerges specifically when there exists a good fit between a person’s skill and the task challenges. Most significantly for this framework, DMN activity is down-regulated by the exploitation mode, which points toward a direct neurochemical pathway for reducing self-evaluative processing during optimal performance states. Van der Linden et al. (2021) further observe that people who experienced flow often report feeling in control, having a clear sense of direction and a condensed perception of time. The latter aspect is understood as a sensation of time flying when the person is in a flow. Such flow-related changes in time perception are hypothesized to be linked to the reduced sense of self. From this perspective, the LC-NE system functions as the modulator of the switch, which acts as the upstream control that regulates whether self-evaluative processing engages based on the demands of the environment.

#### 3.1.2 Step 2. Self-evaluative processing generates characteristic EM field patterns

Not all DMN activity equally sets the boundary conditions for quantum decoherence, as the DMN encompasses multiple subsystems with differing functions. The posterior cingulate cortex and medial prefrontal cortex are specifically implicated in self-referential evaluation, which involves the ongoing assessment of “how am I doing?” that characterizes anxious self-monitoring (Carhart-Harris et al., 2014; Leroy and Cheron, 2020). From the framework’s perspective, this evaluative activity would serve as the primary source of decoherence-accelerating electromagnetic patterns because it would involve generating definite assessments about the self and its states, and these continuous judgments tend toward binary conclusions, which aligns with Busemeyer et al. (2020) finding that binary decisions may be more effective at producing what they termed “collapse” than probabilistic judgments. As proposed, the DMN functions as a boundary-setting mechanism, as it does not interact with quantum processes directly, but it would establish the electromagnetic environment that helps to determine decoherence rates. This distinction between direct interaction and boundary-setting is central to understanding how processes on different timescales can be causally related, as Section 6 will examine in detail.

The posterior cingulate cortex (PCC) appears to play a central role in this process, as Carhart-Harris et al. (2014) report a highly significant finding about the relationship between PCC activity and self-experience. A positive relationship has been found between self-reflection and alpha power (Knyazev et al., 2011) and alpha synchronization during rest and Blood Oxygen Level Dependent (BOLD) activity in regions of the DMN (Jann et al., 2009). Evidence indicates involvement of the DMN in self-reflective and introspective functions (Qin and Northoff, 2011). These researchers recently found a significant positive correlation between the magnitude of alpha power decreases in the PCC after psilocybin and an experience of a disintegration of the individual’s “self” or “ego.” This effect also correlated positively with decreases in delta, theta, beta, and low gamma power, with alpha waves explaining the most variance at 66%. This provides a link between PCC electromagnetic activity (measured through alpha power) and the subjective experience of selfhood. The 66% variance explained by alpha oscillations in a single brain region is an unusually strong correlation, and it points toward PCC activity as a measurable biomarker for the intensity of self-referential processing.

Brodmann Area 10 (BA10) in the medial frontal gyrus is particularly relevant for metacognitive monitoring, as Leroy and Cheron (2020) found that flow emergence required transient hypofrontality, specifically decreased activation in BA10, which is a region involved in metacognitive monitoring.

#### 3.1.3 Step 3. Two pathways from neural activity to microtubules

Neural activity appears to be able to influence microtubule states through two parallel pathways that operate on different timescales and serve different functional roles. Understanding these pathways clarifies how slow DMN fluctuations, operating over seconds to minutes, can influence quantum processes that complete in picoseconds.

##### 3.1.3.1 The fast pathway through direct electromagnetic propagation

Pinotsis et al. (2023) provide evidence that electric fields generated by neurons influence cellular structures down to the molecular level. They put forth evidence for the so-called Cytoelectric Coupling Hypothesis whereby electric fields generated by neurons interact with structures down to the level of the cytoskeleton, which could be due to electrodiffusion and mechanotransduction involving exchanges between electrical, potential and chemical energy. The term associated with this mechanism is ephaptic coupling, which means organization of neural activity and formation of neural ensembles at the macroscale level. This essentially electromagnetic information field propagates to the neuron level, affecting spiking, and down to molecular level interacting with the cytoskeleton, possibly enhancing its information processing capabilities.

Pinotsis et al. (2023) identify three coupling pathways through which electromagnetic fields influence intracellular structures, as ephaptic coupling involves direct field interactions between neurons, electrodiffusion involves ion movement driven by field gradients, and mechanotransduction involves mechanical forces induced by field interactions. They further argue that electrical fields carry information processed by the brain that can organize the brain’s infrastructure to support efficient storage and information processing needed for cognitive flexibility. Consequently, the proposed cytoelectric coupling mechanism connects information at the meso-and macroscopic level down to the microscopic level of proteins such as tubulin, actin and membrane receptors.

Cavaglià et al. (2023) describe in some detail how these electromagnetic fields (EMFs) reach the cytoskeleton stating that at the inner layer, EMFs could transfer information inside the cell by interacting with cytoskeletal structures, such as parallel bundles of microtubules (MTs) in axons and dendrites, which are structurally stabilized by microtubule-associated proteins (MAPs). These EMFs propagate both in the space between neurons leading to inter-neuronal interactions and inside the neuron where they can encode information in the neuronal cytoskeleton. With dipole–dipole interactions propagating at speeds on the order of 10^3^ m/s, the generated electromagnetic fields traverse micrometer distances within neurons over approximately one nanosecond. This fast pathway can, therefore, transmit moment-to-moment electromagnetic patterns generated by neural activity to the microtubule cytoskeleton and it permits current DMN activity to influence coherence conditions within a single pre-firing computation window.

##### 3.1.3.2 The slow pathway through calcium-mediated phosphorylation

Wiest (2025) described MT sensitivity to the electromagnetic patterns produced by neural activity through calcium dynamics stating that MTs can detect the detailed state of the distributed spatiotemporal pattern of electrical activity among populations of neurons via calcium influx that mirrors neural activity and modulates MTs and MAPs. The molecular pathway connecting calcium to microtubules has been characterized in detail by Craddock et al. (2012b) who demonstrated that calcium-calmodulin kinase II (CaMKII), which is activated by synaptic calcium influx during long-term potentiation, directly phosphorylates tubulin proteins in MT lattices. They found that size and geometry of the activated hexagonal CaMKII holoenzyme and the hexagonal lattices in MTs are perfectly matched which permits activated CaMKII holoenzymes to bind to MT surfaces with high affinity additionally enhanced by electrostatic attraction. CaMKII with its 12 kinase domains is activated by the Ca^2+^ influx to post-synaptic neurons and then engages in phosphorylation of microtubules. Craddock et al. (2012b) calculated that CaMKII phosphorylation of tubulin produces electrostatic changes in the range of 6 to 36 kcal/mol per CaMKII-tubulin phosphorylation event, which they describe as encoding that is robust against degradation. CaMKII phosphorylation of MTs specifically interacts with their C-terminal tails, which can exist in multiple states and dynamically oscillate between several conformational states. This process involves interactions with water and ions and hence the resultant conformational changes of C-termini of tubulin alter the local electrostatic environment and potentially influence quantum coherence dynamics within the tryptophan networks discussed in connection with quantum phenomena in microtubules above.

The functional role of this slow pathway differs from the fast pathway. According to Craddock et al. (2012b) the memory information encoded by CaMKII phosphorylation can alter, program and provide a background for MT-based information. The phosphorylation pattern most likely does not carry real-time signals but shapes the energy landscape on which real-time processing unfolds which can involve downstream effects such as MAP attachment patterns, axoplasmic motor protein transport and even integration of inputs to the firing threshold at the proximal axon, and regulation of synaptic plasticity. Notably, this slow pathway operates on millisecond-to-second timescales for activation and can persist over extended periods before dephosphorylation by phosphatases and degradation reverses the pattern. It could encode sustained patterns of neural activity, including chronic anxiety, extended flow states, or learned expertise, into the baseline electrostatic landscape of the microtubule lattice.

##### 3.1.3.3 How the two pathways interact

From this perspective, the fast pathway determines whether quantum processing can occur in the current moment, while the slow pathway shapes the structured landscape that quantum-enhanced parallel exploration navigates. The fast pathway transmits the current DMN state, and within the proposed framework, when self-evaluative activity is high, the electromagnetic environment would become noisier and accelerate decoherence. The slow pathway would encode the accumulated history of past activity, and sustained patterns of high or low self-evaluation become written into the phosphorylation pattern of the microtubule lattice. Together, these pathways converge on the microtubule electrostatic environment, which determines whether and how long quantum coherence can be sustained. This bidirectional architecture is independently supported by Plankar et al. (2013), who reviewed evidence that the intraneuronal matrix of cytoskeletal filaments and their associated counterion atmospheres functions as an electrically active network that both receives modulatory input from neural activity through calcium signaling and feeds back to modulate membrane excitability and ion channel gating through ionic wave propagation along filament surfaces.

#### 3.1.4 Step 4. Microtubule environment determines quantum coherence

The experimental evidence reviewed in Section 2 established that microtubules support quantum coherent energy migration, as Kalra et al. (2023) demonstrated that microtubule tryptophan networks exhibit quantum energy migration with diffusion lengths of approximately 6 nm and decoherence times on the order of 5 ns. Subsequently, Babcock et al. (2024) found quantum yield enhancements of up to 70% compared to isolated tryptophan molecules, with size-dependent robustness where larger systems actually become more stable.

This paper’s framework proposes that this quantum coherence is modulated by the electromagnetic and ionic environment generated by neural activity through both pathways described above. Within this framework, higher self-evaluative activity generates stronger electromagnetic patterns through the fast pathway and more phosphorylation activity through the slow pathway, which together create noisier ionic environments that accelerate decoherence and push the system toward classical processing. Lower self-evaluative activity creates more stable conditions through both pathways, which permits extended coherence and quantum processing. This creates a tunable parameter that links neural activity patterns to quantum dynamics in microtubules, with transient modulation through electromagnetic propagation and cumulative modulation through phosphorylation history.

**Figure 1:**
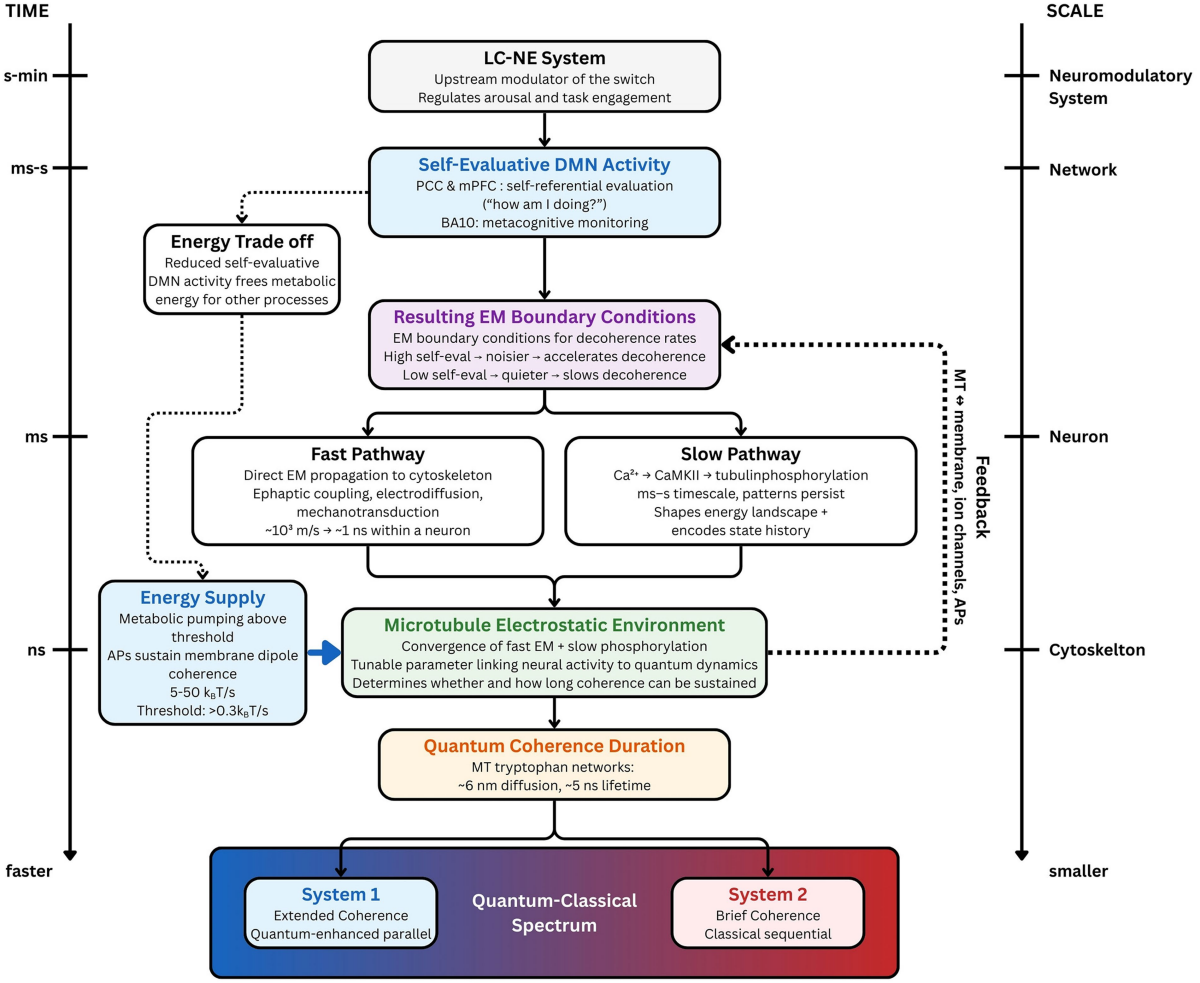
The proposed causal chain from self-referential processing to cognitive mode. The mechanism operates across multiple orders of magnitude in time (left axis) and spatial scale (right axis). The LC-NE system (Box 1) regulates self-evaluative DMN activity (Box 2), which generates electromagnetic boundary conditions (Box 3) that influence microtubule states through two parallel pathways: a fast pathway (~1 ns) via direct electromagnetic propagation and a slow pathway (ms–s) via calcium-mediated phosphorylation. These pathways converge on the microtubule electrostatic environment (Box 5), which determines the duration of quantum coherence (Box 6). Extended coherence biases processing toward System 1 (quantum-enhanced parallel processing), while brief coherence biases processing toward System 2 (classical sequential processing). Metabolic energy supplied via action-potential-driven membrane activity (blue box, left) must exceed a threshold (>0.3 k_B_T/s) for coherence to be sustained. Reduced self-evaluative DMN activity frees metabolic resources for coherence maintenance (dashed arrow, left). Bidirectional feedback exists between microtubule properties and membrane dynamics (dashed arrow, right).

It was also experimentally determined and computationally elucidated (Tuszynski, 2024) that MTs possess transistor-like (Priel et al., 2006) and memristor-like (Tuszynski et al., 2020) properties that could be involved in modifying the interactions between microtubules and motor proteins, the membrane, ion channels and even action potentials. Mohsin et al. (2025) independently characterized the molecular mechanism underlying these properties through their electrokinetic model, showing that nanopore-mediated ionic exchange between the inner and outer microtubule surfaces produces self-sustaining leapfrogging solitons that oscillate at approximately 39 Hz, a frequency within the gamma band associated with cognitive processing (Kounios and Beeman, 2009, 2014). This is consistent with a bidirectional relationship where membrane activity influences microtubule states and microtubule properties influence membrane dynamics, which could create a feedback loop that could amplify or dampen quantum coherence based on the overall state of the system (see Figure 1).

The magnitude of this proposed effect in living neural tissue has not been directly measured, which is one reason the preceding and following sections assemble convergent evidence from independent research programs. Pinotsis et al. (2023) provide evidence that endogenous electromagnetic fields reach the cytoskeleton, Kalra et al. (2023) demonstrate that changes in the local dielectric environment alter microtubule quantum properties by 12%–15%, and the anesthesia evidence reviewed in Section 4 supports the proposal that disrupting microtubule quantum dynamics is sufficient to abolish consciousness. The energy analysis in Section 5 then quantifies the metabolic constraints within which this pathway would operate, and Section 7 examines whether the cognitive phenomena predicted by the mechanism appear in the empirical record. The framework’s contribution is to identify these independent findings as components of a single pathway and to generate the testable predictions needed to characterize the quantitative relationship between self-evaluative electromagnetic activity and microtubule decoherence rates.

## 4 Mechanism of anesthesia

The mechanism proposed in Section 3 generates a specific prediction about anesthesia. If waking conscious experience depends to some degree on quantum-coherent information propagation within microtubules, then a natural question arises about what would happen if that coherence were disrupted. Anesthesia presents an opportunity to examine this because anesthetics reliably and reversibly abolish consciousness while leaving the organism alive, and as Wiest (2025) has analyzed in detail, this selectivity raises the question of what specifically is being targeted.

The evidence from anesthesia research therefore provides an independent test of the framework’s core mechanistic claims. Inhalational anesthetics bind promiscuously at hydrophobic pockets in a variety of proteins in the brain and spinal cord (Eckenhoff and Johansson, 1997) which was demonstrated by detailed computational studies for tubulin (Zizzi et al., 2022) and actin (Truglia et al., 2023). In general, it is, therefore, currently believed that anesthetic molecules cause unconsciousness by acting on a combination of ion channels and receptors, synaptic proteins and gap junctions, mitochondria, and cytoskeletal proteins including microtubules. However, Wiest (2025) identifies multiple empirical facts that strongly suggest that these diverse anesthetic compounds act primarily on a single highly conserved molecular target protein which causes a selective abolition consciousness. He points first to the Meyer-Overton correlation, which was established over a century ago and shows that anesthetic potency correlates with solubility in olive oil across several orders of magnitude (Katz, 1994). This correlation is believed to indicate that anesthetics interact via weak physical interactions such as van der Waals forces rather than ionic or covalent bonding. Moreover, according to Wiest (2025) while anesthetics target multiple molecular targets leading to various associated effects (e.g., analgesia and amnesia), a primary unitary molecular target for the loss of consciousness might be more plausible than a varying combination of ion channels and other targets. Interestingly, the minimum alveolar concentration (MAC) of a given anesthetic varies little across diverse species despite broad variability of the structures of different ion channels in animals. Consequently, no single ion channel can account for the loss of consciousness caused by inhalational anesthetics, and no combination of ion channel targets can account for the pattern of empirical results accumulated in the literature.

### 4.1 Microtubules as a convergent molecular target of anesthesia

Craddock et al. (2017) conducted quantum modeling studies that predicted anesthetic potencies based on their effects on the quantum energy levels of tubulin in the binding sites for several anesthetics, which essentially reproduce the Meyer–Overton correlation by assuming that anesthesia is primarily mediated by MTs. This cannot be said for any other candidate molecular target which is consistent with microtubules serving as the primary molecular target that mediates the loss of consciousness due to the action of inhalational anesthetics. The quantum modeling identified specific binding sites where anesthetics interact with the aromatic electron clouds in tubulin which overlap with tryptophan networks that Kalra et al. (2023) and Babcock et al. (2024) have shown to support quantum coherent energy transfer.

In fact, Kalra et al. (2023) additionally provided direct experimental evidence that anesthetics affect microtubule quantum properties. They found that the presence of etomidate and isoflurane in the solution containing microtubules decreased the exciton diffusion lengths from 6.6 ± 0.1 nm to 5.6 ± 0.1 nm and 5.8 ± 0.2 nm, respectively, representing a reduction of approximately 12%–15% in the quantum energy migration efficiency within microtubule tryptophan networks. Kalra et al. (2023) team indicated that these observations suggest that etomidate and isoflurane dampen energy transfer between tryptophan residues in tubulin due to changes in the dielectric screening of electronic interactions. In essence, these anesthetics alter the local electromagnetic environment surrounding the tryptophan chromophores, which degrades the quantum coherent energy transfer within their network. Consequently, this finding establishes a direct link between anesthetic action and microtubule quantum properties as the compounds that reliably eliminate consciousness in clinical settings demonstrably impair quantum energy migration in microtubules.

Additional evidence for microtubules as a primary anesthetic target comes from intervention studies as Khan et al. (2024) administered brain-penetrant microtubule-binding drugs to rats before exposing them to the volatile anesthetic isoflurane. They found that rats given the microtubule-binding drugs took significantly longer to become unconscious under the influence of isoflurane. If microtubules were merely incidental to anesthetic action, then protecting them by binding of drugs to tubulin should have no effect on the onset of unconsciousness, so the observation that microtubule protection actually delays unconsciousness supports a causal relationship between microtubule function and conscious states.

## 5 Energy-based mechanism

The switch between System 1 and System 2 processing as proposed here is fundamentally a switch in energy allocation within the human brain. Considering this, quantum coherence would be less like a state the brain possesses and more like a process that the brain sustains with continuous metabolic work, because each moment of maintained coherence incurs a cost in terms of biochemical energy units, ATP molecules. Hence, when energy pumping falls below a critical threshold, quantum state coherence would decay to a thermal equilibrium.

Quantum coherence in biological systems requires continued energy input, and Cavaglià et al. (2023) argue that neuronal membranes can satisfy the conditions for Fröhlich’s quantum condensate formation, potentially generating coherent electromagnetic fields. They identify the following five criteria for the coherence within the dipole moments present in the phospholipid head groups of the neuronal membrane:

(a) A sufficient supply of metabolic energy above a minimum threshold required to achieve synchronization of membrane dipole oscillations.
(b) The presence of thermal noise due to physiological temperature.
(c) Internal structural organization facilitating strong interactions between dipoles.
(d) The existence of a large trans-membrane electric potential difference.
(e) Nonlinear interactions between oscillating head group dipoles.

They established by mathematical estimates that the above conditions are satisfied by the neuronal membrane phosphate groups’ dipoles provided both action potential energy (proportional to the electric potential gradient) and the rate of AP generation is sufficiently high. The strength of electromagnetic fields generated by neuronal membranes is substantial, as Cavaglià et al. (2023) report that the membrane’s strong electric potential gradients on the order of 100 mV across the thickness of 5 nm yielding electric field intensities reaching 20 × 10^6^ V/m (i.e., 20 million volts per meter).

The dipole–dipole interactions are also significant relative to thermal noise, since the dipole moment of each phosphate head group is approximately 20 debye, so the dipole–dipole interaction, assuming a 5-angstrom distance between the centers of these dipoles is on the order of 9 k_B_T or even higher since each group has several neighbors. Cavaglià et al. (2023) calculated the rate of energy pumping, S, knowing the rate of arrival of APs as being in the range of values given by the inequality: 5 k_B_T/s < S = E_ap-d_/dt < 50 k_B_T/s, which easily exceeds the minimum requirement of 0.3 k_B_T/s for a so-called weak Bose-Einstein condensate determined computationally in an earlier publication (Reimers et al., 2009).

Given these conditions, the predicted coherent modes of dipole oscillations are in the 10^11^–10^12^ Hz frequency range consistently with the original calculations by Fröhlich (1977). These dipolar oscillation waves are expected to propagate with a velocity of about 10^3^ m/s, much faster than AP propagation that ranges between 0.5 and 10 m/s. Since Maxwell’s theory of electromagnetism shows that oscillating dipoles emit EMFs, coherently oscillating dipoles of axonal membranes due to (incoherent) energy input from APs, are capable of EMF generation in the process. Their detection and the measurements of their intensity and dispersion relations are open to investigations.

The transition from quantum to classical behavior is understood in modern decoherence theory as a gradual process. Zurek (2003) work on quantum decoherence and the transition to classicality established that environmental interaction progressively destroys quantum superpositions, with the rate depending on the strength and nature of system-environment coupling. Melkikh (2015) extends this view, as he argues that collapse to a point can never happen while increasing temperature causes the interference pattern to gradually disappear. Donadi et al. (2021) support this gradient perspective. This gradient view is important for the present framework because if collapse were an all-or-nothing transition, then biological control would be difficult to achieve, as the system would either be fully quantum or fully classical with no intermediate states. If collapse occurs along a gradient governed by environmental interaction strength, then modulating the internal electromagnetic environment could shift the position of neural systems along the quantum-classical spectrum without requiring perfect environmental isolation (Dutton et al., 2004; Hau, 2016), which is what the DMN-mediated mechanism proposed in Section 3 would accomplish.

The DMN’s energy consumption provides context for better understanding this allocation. Carhart-Harris et al. (2014) stated that DMN regions receive more blood flow and consume more energy than other brain regions, with metabolic rate approximately 40% higher in the PCC than the brain average. From the present framework’s perspective, this energy differential can be explained by the need to sustain the electromagnetic activity that may maintain the boundaries of the ego and its states. These boundaries are adaptive for survival, as they enable definite self-other (subject-object) distinctions and rapid threat assessment, but the same electromagnetic activity sets the boundary conditions that accelerate quantum decoherence in microtubules, so reducing DMN activity would free metabolic resources for the maintenance of coherence in other areas, which suggests there is a fundamental trade-off between these two modes. Gallimore (2015) reaches a complementary conclusion using Integrated Information Theory, demonstrating mathematically that a very low entropy state would maximally constrain past and future states of the brain and generate a highly inflexible state of consciousness, while excessively high entropy would maximize the number of available states with a reduced ability to constrain outcomes. From this perspective, the brain continuously navigates the allocation of metabolic resources between competing computational modes.

### 5.1 Anesthesia as energy supply impairment

The mechanism of anesthesia based on the description in Section 4 becomes better understood when viewed through an energy lens, and it sheds light on the energy consumption that Carhart-Harris et al. (2014) outlined. Winegar and MacIver (2006) recorded hippocampal neural activity under isoflurane and found that at clinical concentrations of about 1 MAC, isoflurane depressed glutamatergic EPSPs by roughly 60 % while paired-pulse facilitation increased, which points toward a presynaptic reduction in excitation-release coupling. Fiber volleys, by contrast, were found to be strongly resistant to isoflurane and somatic spike currents were also unaffected by 350 μM (1 MAC) isoflurane.

This has valuable implications as axonal action potentials continue to propagate under anesthesia (although at lower rates and intensity amplitudes), and basic neural signaling persists, but what becomes impaired by anesthesia is synaptic output and local energy transfer at terminals. From the present framework’s perspective, this pattern fits an energy-based account as action potentials carry electric fields and ionic fluxes that pump energy into the membrane-microtubule system, and when their rate and synaptic effectiveness fall under anesthesia, the net energy supply for maintaining microtubule coherence and Fröhlich-type oscillations drops below the threshold required to resist thermal decoherence. In this energy environment, waking consciousness then fades even while basic spike conduction persists.

Wiest (2025) articulates a strong version of the quantum hypothesis for anesthesia, as he proposes that anesthetics disrupt large-scale coherent quantum states in microtubules. This was previously hypothesized by Craddock et al. (2012a) and later linked to specific changes in the quantum states of tubulin in microtubules (Craddock et al., 2017).

From this perspective, the specificity of anesthetics for consciousness makes sense even though these compounds bind promiscuously to many proteins throughout the body. Quantum coherent states are fragile and require precise coordination, while incoherent quantum processes are already randomized and insensitive to further randomization. Anesthetics would selectively disrupt the orchestrated quantum activities that support consciousness while still leaving other cellular functions intact. The rapid onset and reversibility of general anesthesia place an additional constraint on the mechanism, because the transitions that abolish unified conscious experience within seconds may be difficult to reconcile with gradual synaptic or network reconfiguration, but they may be consistent with energy-threshold-dependent loss of coherence in a system that operates near criticality, where slight perturbations can rapidly shift the system between ordered and disordered regimes.

This quantum hypothesis also accounts for the observation that microtubules can function to detect and respond to the direction and orientation of electromagnetic signals (Wiest, 2025), which was initially uncovered by G. Albrecht-Buehler in connection with the functioning of centrosomes as the so-called “eyes of the cell” (Albrecht-Buehler, 1994). Microtubules (or their aggregates such as centrioles) appear to function as cellular sensors that integrate environmental information, and this sensory function may depend on quantum coherent properties that anesthetics disrupt by reducing the amount of energy MTs receive.

### 5.2 Energy thresholds

Considering what the paper has covered so far, anesthesia would likely push the system below the coherence threshold through two converging mechanisms. First, the reduced synaptic effectiveness demonstrated by Winegar and MacIver (2006) decreases the net energy available for membrane coherence. Second, as Kalra et al. (2023) showed, anesthetics directly bind to microtubule tryptophan networks and reduce quantum energy migration efficiency. The combination of a reduced energy supply and the impaired quantum infrastructure would gradually transition the system from a coherent to incoherent regime.

A noteworthy feature of volatile anesthetics is their effectiveness across the entire tree of life. As noted by Wiest (2025) the same anesthetic molecules used on human patients also reversibly slow or halt motility in single-celled organisms and even plants. This cross-kingdom effectiveness is difficult to explain if anesthesia operates through specific ion channel mechanisms, because as mentioned above, ion channel profiles vary widely across species and single-celled organisms lack nervous systems entirely. Wiest notes that this is what caused Claude Bernard to say, “What is alive must sense and can be anesthetized. The rest is dead.” From an energy-based perspective, any system that relies on sustained energy pumping to maintain quantum coherence would be vulnerable to disruption by compounds that either reduce energy supply or directly impair the quantum infrastructure, regardless of whether that system has neurons.

Considering this, as an analogy for this threshold-dependent behavior, the transition between conscious and unconscious states under anesthesia may resemble the transition between a laser and a lamp. A laser maintains coherent light emission only while being pumped above a threshold, but below this threshold, the same atoms emit incoherent thermal light, while the physical substrate is unchanged. The proposed brain analog would include metabolic energy pumping via action potentials, Fröhlich condensates of coherent membrane dipole oscillations, superradiant emission through microtubule tryptophan networks (Cavaglià et al., 2023; Babcock et al., 2024; Nishiyama et al., 2024a), and feedback via electromagnetic field-cytoskeleton interactions (Cavaglià et al., 2023; Pinotsis et al., 2023). It should also be mentioned that the energy of photoexcitations is very high compared to thermal noise present due to physiological temperature providing robustness of interactions a high signal-to-noise ratio.

### 5.3 Thermodynamic constraints on computation

Kurian (2025) identifies fundamental thermodynamic constraints that govern biological computation by stating that entropy limits memory, and energy limits speed. These constraints would shape the trade-offs between quantum and classical processing modes and help explain why the allocation of metabolic energy between coherence maintenance and self-evaluative processing has computational consequences.

The physics of information erasure places hard limits on the energy cost of computation as Landauer (1961) showed that discarding one bit of information requires a minimum energy expenditure of k_B_T ln 2, which established that information processing has unavoidable physical costs. Bérut et al. (2012) confirmed this experimentally and Jun et al. (2014) extended these findings by comparing two protocols that were physically nearly identical but differed in whether they preserved or discarded information about initial conditions. According to Jun et al. (2014) the full-erasure protocol involving the compression of phase space from two states to one, asymptotically requires k_B_T ln 2 of work, while a very similar no-erasure protocol, which has no such phase-space compression, is reversible hence incurring no energy cost. This asymmetry between preserving and discarding information has implications for how biological systems allocate computational resources.

Applying this to the present framework, the DMN’s substantial energy consumption becomes more understandable as each binary self-assessment, of the kind whose effects on subsequent processing were measured by Kvam and Pleskac (2017) and Busemeyer et al. (2019, 2020) would commit information and discard alternatives, so maintaining coherent self-boundaries would require continuous collapse of possibilities into definite states, with each collapse carrying thermodynamic cost. The 40% higher metabolic rate in the PCC that Carhart-Harris et al. (2014) reported may reflect this energetic burden of sustaining definite self-states.

Interestingly, Troubat et al. (2009) studied elite chess players during competition and found that total energy expenditure increased only about 10 percent above rest, which is far less than classical computational models would predict when evaluating billions of possible moves under strict time constraints. This efficiency aligns with the thermodynamic framework, as expert cognition operating in a regime of extended coherence with late collapse would avoid the continuous Landauer costs associated with premature commitment. These energy constraints mean that biological systems cannot achieve perfect reversibility, but they can still operate far more efficiently than architectures that commit early and often.

Considering this thermodynamic perspective, the trade-off between System 1 and System 2 processing may take on physical grounding as System 1, which preserves indeterminate states and delays commitment, would operate in a much more thermodynamically efficient regime where possibilities are maintained. System 2, which commits early to definite states for serial manipulation, would incur the Landauer cost at each step. The brain’s allocation of resources between maintaining superposition and forcing collapse would be governed by task demands and also by the metabolic costs of each computational mode which are costs that arise from the physics of information itself.

### 5.4 Quantification of the brain’s energetic demands for information storage and processing

The preceding sections established that the brain operates under energy-dependent coherence thresholds, that self-evaluative DMN activity consumes a substantial share of the neuronal energy budget, and that even modest perturbations in energy supply can shift the system between coherent and incoherent regimes. What has not yet been established is a quantitative picture of the energy budgets themselves. How much processing power does the brain have available at each level of its structural hierarchy, and what are the physical limits on information processing given these metabolic constraints? Patwa et al. (2024) concluded that classical energy models could not account for the brain’s computational efficiency at only approximately 20 W of input power, and Kurian (2025) estimated that classical Hodgkin-Huxley mechanisms process approximately 10^3^ operations per second per neuron while superradiant protein fibers may reach approximately 10^13^. If these estimates are correct, the question becomes how the brain allocates its limited energy budget across the many levels of its processing hierarchy, and whether the energy constraints themselves favor the kind of hierarchical and threshold-dependent architecture proposed in the preceding sections. The analysis that follows performs physics-based estimation (Tuszynski, 2018) of metabolic energy consumption and maximum information-processing rates at each scale, with the goal of producing order-of-magnitude bounds. The analysis proceeds at a classical physics level to establish baseline energy constraints that any processing mode, whether classical or quantum, must respect.

Stringent limits on information storage and processing arise directly from the brain’s metabolic constraints (Laughlin et al., 1998; Laughlin, 2001; Laughlin and Sejnowski, 2003). The human body operates at roughly 100 W of total power, and this energy must be distributed across approximately 3.7 × 10^13^ cells, including the brain’s approximately 10^11^ neurons, each embedded in a dense network of roughly 10^4^ synaptic connections (Nicholls et al., 2001). At the cellular level, this global constraint translates into extremely limited per-unit energy availability, as the average power per cell is on the order of 3 × 10^−12^ W. At this scale, even modest redistributions of metabolic resources between competing processes could shift the energy available for any single function, which is consistent with the sensitivity of coherence thresholds discussed in the preceding sections. Sustaining even this baseline requires the continuous production of roughly 10^21^ ATP molecules per second, which reflects a near-complete daily turnover of the body’s ATP mass. This energy is generated through oxidative phosphorylation within mitochondria, of which there are approximately 10^3^ per cell and roughly 3.7 × 10^16^ in the body as a whole. Each mitochondrion produces on the order of 3 × 10^4^ ATP molecules per second (Aon et al., 2006), where one molecule of glucose yields 38 molecules of ATP, and given that each ATP synthase operates at approximately 600 ATP molecules per second, this implies roughly 50 ATP synthase enzymes per mitochondrion. The result is a tightly constrained and highly distributed energy supply at the scale of individual computational elements.

Among all organs, the brain has a disproportionately high rate of energy consumption, as it uses roughly 25% of the body’s total metabolic demand, or about 25 W on average. ATP molecules provide the vast majority of this energy, with each molecule releasing approximately 10 k_B_T (where k_B_ is the Boltzmann constant), equal to 4 × 10^−20^ J. Although the brain’s primary function involves storing and processing sensory information, the large majority of its energy budget, approximately 70 to 80%, goes toward thermoregulation. Much of the remaining energy supports the protein synthesis machinery, including ribosomes, as well as protein repair and removal. A conservative estimate places the fraction of brain energy devoted to information storage and processing at less than 5%, or approximately 2.5 W. Distributed across roughly 10^11^ neurons and a comparable number of glial cells, this yields an average power demand of approximately 1.2 × 10^−11^ W, or 12 pW, per neuron. This figure carries weight because information encoding and erasure both carry energetic costs, and translating power demands into the energy budgets of individual processes requires knowing the relevant time scales, which can span many orders of magnitude.

All forms of information carry an energetic cost. Landauer (1961) established that the minimum energy required to encode or erase one bit of information is *ε* = kT ln (2) = 4 × 10^−21^ J, a result that follows from the thermodynamic relationship F = U − TS, where entropy S is equivalent to negative information, −I. Landauer (1996) extended this framework by treating information as a fundamentally physical quantity, and Shannon’s formula I = −kT ln Ω, with Ω representing the number of equiprobable states available for information storage, defines the informational content of a given ensemble. This value ε sets the theoretical floor for the energetic cost of creating one bit of information. In biological systems, the actual energetic cost of information processing is expected to exceed this theoretical minimum, as the energy released by a single ATP molecule is approximately an order of magnitude greater. In order to relate metabolic energy expenditure to the information-processing rate and estimate its maximum value, the time scale of the predominant bit-switching processes, Δ*τ*, must be determined. For the entire brain, the rate of information processing can be estimated as shown in Equation 1:


$$\frac{\mathrm{\Delta I}}{\Delta \tau }=\frac{P}{\varepsilon }=\frac{2.5\;W}{4\times 10^{-21}\;J}=6\times 10^{20}\;\text{bits}/s$$ (1)

This figure establishes an absolute upper bound on what the brain could achieve computationally within a classical mode of operation. Because the energetic requirements of quantum processes in neural tissue are not yet well characterized, this classical upper bound also serves as a working estimate for the brain’s overall information-processing capacity.

Maximum processing rates at each level of the hierarchy are constrained by the characteristic time scales of the brain’s information-processing units and by the limited power available to each neuron. Libet et al. (1979) determined the pre-processing time of the human brain to be approximately 500 ms, and it can be inferred that for the brain as a whole, the characteristic time scale Δt is on the order of 1 s or less, which is consistent with EEG-recorded brain wave frequencies in the 10 to 100 Hz range. At this scale, the brain’s maximum information-processing rate of 6 × 10^20^ bits/s is enormous compared to even the largest supercomputers. Moving to the next level of the structural and functional hierarchy, there are approximately 10^11^ neurons in the brain, and if all neurons are assumed to contribute equally, a single neuron would be expected to process on the order of 6 × 10^9^ bits/s, or approximately one gigabyte per second. Neuronal firing rates commonly occur on the order of 1 ms, which places the corresponding frequency in the 1 kHz range and would allow a neuron to hypothetically process as much as one megabyte per ms. This corresponds to a single information package transmitted per processing event.

If microtubules serve as the computational elements within neurons, with approximately one thousand (10^3^) microtubules per neuron, the average processing rate per microtubule would be approximately 10^6^ bytes/s, or one megabyte per second. The corresponding time scales for microtubule processing are on the order of 1 μs, which is typical for a conformational change in a protein, and the corresponding frequency would be approximately 1 MHz. At this level, a single conformational change event can only encode one byte of information. Moving to the lowest level of this hierarchy, the constituent protein tubulin must be considered. A typical 10 μm-long microtubule contains on the order of 10^4^ tubulin dimers, and if each tubulin dimer serves as the basic computational element corresponding to a bit of biological information, its maximum information-processing rate would be approximately 100 bytes/s.

This estimate creates a tension with proposals that treat tubulin as a biological qubit operating on nanosecond or sub-nanosecond timescales. Reconciling these timescales with the energetic constraints would require either that individual information-processing events occur very infrequently, on the order of once every 10 million nanoseconds, or that only a small fraction of tubulin dimers, perhaps one in 10 million, are engaged in continuous information encoding at any given moment. Post-translational modifications of tubulin can encode biological information (Janke and Kneussel, 2010), but this process operates too slowly to support memory formation, and the question of how specific tubulin dimers would be selected for processing remains open.

The purpose of the analysis in this section is to establish the energy constraints that any processing mode must respect, and the estimates presented here deliberately proceed under classical assumptions in order to identify the tightest possible bounds. It is worth noting that the tension identified above assumes tubulin operates as a classical bit, and if tubulin dimers instead function as coupled qubits whose collective quantum states carry information in amplitude and phase relationships across the lattice, the energy cost per unit of information would change substantially, as reversible quantum evolution does not incur Landauer costs until collapse occurs. Recent trajectory-based analyses of quantum-to-classical transitions in biological light-harvesting systems have demonstrated that coupled two-level systems can sustain coherence through environment-dependent dynamical corridors on the Bloch sphere (Uthailiang et al., 2026), and similar formalism applied to the microtubule lattice could potentially resolve the tension identified here. The present analysis does not take a position on this question, but it establishes the classical energy constraints against which future quantum-level analyses can be evaluated. The switching mechanism proposed in this paper operates at the level of energy allocation and electromagnetic boundary conditions, which means it is compatible with either interpretation. Whether tubulin operates as a classical bit or as a coupled qubit would have significant implications for the computational capacity available to System 1 during periods of extended coherence.

Considering these estimates, the conclusions point toward the possibility of state changes due to information encoding on the order of tens of milliseconds. Microtubule coupling to action potentials propagating along the neuron’s axon could mechanistically account for this timescale, but sub-nanosecond electronic transitions and GHz frequency rates would not be compatible with these energy budgets. If information processing takes place in the brain in a hierarchical manner, with neurons forming the second layer, microtubules the next, and selected tubulin dimers contributing at the finest scale, the maximum theoretically possible information-processing rates would be approximately (a) 10^20^ bytes/s per brain, (b) 10^9^ bytes/s per neuron, (c) 10^6^ bytes/s per microtubule, and possibly, with open questions about long-term stability, (d) 10^2^ bytes/s per tubulin. These figures may reasonably quantify the biochemical processes occurring in the brain within the classical regime, and they are consistent with multiple biochemical scenarios. Phosphorylation events of tubulin by CaMKII (Craddock et al., 2012b), could possibly occur on timescales of tens of milliseconds, and interactions of microtubules with action potential fields due to ionic waves crossing the axonal membrane, which propagate at speeds on the order of 1 m/s, would operate in a comparable range.

The estimates presented in this section establish energy bounds within which both classical and quantum processing must operate. As reviewed in Section 2, recent experimental work has demonstrated that microtubule tryptophan networks support quantum coherent energy migration at physiological temperatures (Kalra et al., 2023; Babcock et al., 2024), with superradiant emission operating approximately two orders of magnitude faster than thermal decoherence effects (Nishiyama et al., 2024a). The energetic analysis here complements those findings by establishing that if the very rapid transition rates associated with quantum states of tubulin (Mershin et al., 2004) were operating continuously across all tubulin dimers, the corresponding power consumption would amount to tens or hundreds of megawatts, which is clearly an unrealistic value in the context of the human brain. The speed of information processing is therefore limited by both the amount of energy provided to each neuron and by the time scales on which individual states encoding biological information can change. This constraint does not preclude quantum processing, but within the classical energy framework used here, it does require that quantum events occur infrequently or involve only a small fraction of available substrates at any given moment, which is consistent with the hierarchical and threshold-dependent architecture proposed in the preceding sections.

A parallel line of reasoning can be applied to ion channels. There are on the order of 10^4^ ion channels per neuron, which places the maximum information-processing rate per ion channel at approximately 10^5^ bytes/s, a number that is consistent with the brain’s estimated power consumption. This translates into a time scale of 10 μs for information-processing events involving each ion channel. In model channels like the bacterial KcsA channel, one K^+^ ion crosses the channel per 10 to 20 ns under physiological conductances of roughly 80 to 100 pS (Roux and Schulten, 2004), which allows for a maximum conduction rate of approximately 10^8^ ions/s. The distance between the center of the channel pore and the membrane surface can be estimated at approximately 5 nm, and a continuum electro-diffusion model (Allen et al., 2000; Guidoni and Carloni, 2002) would provide an average ion speed of approximately 0.5 m/s. This model does not account for the ion-channel structure, masses, and thermal energies, or for movement within the filter itself. A more accurate estimate places the time for a single ion to traverse a 5 nm ion channel at approximately 5 ps, which gives an average speed of about 1,000 m/s. The corresponding kinetic energy together with the electrostatic potential energy amounts to approximately 2 × 10^−20^ J, which closely matches the energy of a single ATP molecule and justifies the active transport requirement. The ion flow rate per channel is on the order of 10^5^ ions/ms, which gives a clocking time of approximately 10 ns, and a 5 ps active event is therefore separated by a refractory interval approximately 2,000 times longer.

Because the transmembrane potential is on the order of 100 mV, a flow of singly charged ions like sodium or potassium produces a current on the order of 10 pA, and the corresponding frequency range is approximately 2 × 10^11^ Hz. For a single ion transition across the channel, the energy involved in this oscillation is E = hf ≈ 1 meV, which falls below the thermal energy of k_B_T. If the ion channels of a neuron are synchronized, the total energy value of coupled ion channel transitions could reach up to 4 eV (Bernèche and Roux, 2001), which is well within the quantum range and corresponds to the energy of a photon at approximately 310 nm in the UV wavelength range. The non-conducting state persists for approximately 10^−3^ to 10^−4^ s, and within the selectivity filter each translocation event takes on the order of 10^−11^ s, which means approximately 10^7^ filter state changes can occur during a single non-conducting interval, and approximately 10^10^ switches per second (10 GHz) would represent the fastest information-encoding rate that remains consistent with the energetic and physiological limitations discussed above.

Within the framework proposed in this paper, microtubules have been identified as the primary substrate for quantum coherent processing that supports System 1 cognition. The energetic analysis presented here also indicates that ion channels may play a complementary role, as both cytoskeletal proteins and ion channels (Karplus and McCammon, 1983) appear to be feasible candidates for quantum information processing (Matsuno and Paton, 2000) given the time and energy scales involved. The order-of-magnitude analysis indicates that coupled and synchronized ion channels (Liu et al., 1990; Tsong, 1990; Traub et al., 2002) within a single neuron may collectively generate oscillations in the quantum energy range, with ion channels potentially contributing to rapid information processing while microtubules serve as the primary substrate for quantum coherent computation and information storage. Plankar et al. (2013) reach a similar conclusion, noting that both cytoskeletal proteins and ion channels form a functionally integrated system within the intraneuronal matrix, where coherent dynamics in one substrate can modulate the other through counterion-mediated electric signaling and direct structural coupling.

The consistency of frequency and length scales across the various levels of this information-processing hierarchy provides further support for these conclusions. In a lattice-like model (Sahu et al., 2013), the distance between synchronized neighboring elements (Draguhn et al., 1998) sets the characteristic length scale, and the inverse of the characteristic time yields the corresponding frequency. Multiplying these two quantities produces the propagation velocity of a collective excitation. Atoms in a protein are separated by angstrom distances and vibrate on femtosecond time scales, which gives propagation velocities in the range of 10^4^ to 10^5^ m/s. Protein–protein distances within a microtubule, for example between neighboring tubulins, are in the 10^−8^ m range with characteristic times in nanoseconds, which leads to propagation velocities of 10 to 100 m/s. Microtubule-to-microtubule interactions in an axonal bundle occur across distances of approximately 100 nm and on time scales of fractions of microseconds, which gives rise to velocities on the order of m/s that can couple with action potential propagation. Ion channels can be separated by distances of tens to hundreds of nm with characteristic time scales on the order of 10 ns, which also gives rise to propagation velocities between m/s and tens of m/s (Kuyucak et al., 2001).

For a mechanistic picture of ion channel information propagation, consider a single ion located inside the selectivity filter of an ion channel (Bernèche and Roux, 2005) and assume that it encodes 1 bit of information as proposed by Bernroider and Roy (2004, 2005). With a state transition from outside to inside the neuron, the corresponding bit-switching rate amounts to 10^10^/s. Assuming 10^4^ channels per cortical neuron, an ensemble of roughly 10^6^ neurons engaged in a single perception event would perform a maximum of 10^10^ × 10^4^ × 10^6^ = 10^20^ bits/s, which is comparable to the upper limit of the brain’s processing rate as evaluated above. If a comparable calculation is applied to each of the five senses engaged in communication with the outside environment, the information-processing rate would approach the limit of 6 × 10^20^ bits/s, and only a fraction of the brain’s available metabolic energy would remain for other types of continuous computation.

Neuron-to-neuron distances across synaptic connections are in the micron range while typical interaction time scales are on the order of milliseconds, which leads to synchronization propagation speeds in the mm/s range. This number, considered alongside Libet’s pre-processing time estimate, may help to explain the limited size of specialized domains in the brain, which also corresponds to the resolution limit of modern fMRI equipment. Sahu et al. (2013) proposed a hypothesis of hierarchical resonant fractal functioning in the human brain, which may account for the presence of different clocking rates at different spatial scales and the resonances that connect them. This framework could also shed light on how information is filtered as it passes from molecular to cellular to organ-level scales, with increasing coarse-graining at each level of the hierarchy. According to Sahu et al. (2013), the brain has a total bandwidth spanning from 10^−15^ to 10^15^ Hz, and memory can be stored in a fractal manner across every level.

Based on this analysis, a multi-fractal hypothesis can be proposed because the scaling laws connecting different information-processing hierarchies are not identical. Table 1 presents the hierarchical organization, and the scaling exponents that link neighboring elements differ at each level, which supports an architecture where different computational regimes operate at each scale.

**Table 1:** Hierarchical organization of information processing elements in the human brain with their numbers, characteristic frequencies (in Hz), and scaling exponents linking neighboring elements in the hierarchy.

Element	Number *N*	Characteristic frequency *f*_1_	*F_N_*	Scaling exponent *α*: (*f*_N_) ~ (*f*_1_)^a^
Neuron-brain	10^11^	10^4^	10^1^	1/4
MT-neuron	10^3^	10^7^	10^4^	4/7
Ion channel-neuron	10^4^	10^8^	10^4^	1/2
Tubulin-MT	10^4^	10^9^	10^7^	7/9

A hierarchical organization of this kind, with different time scales and frequency scales at each level, offers a potential resolution to the question of how the brain’s limited energy budget can support quantum processing. Within the framework proposed in this paper, information-processing events that are extremely fast, on the order of 1 fs in duration, could occur with long temporal separations on the order of milliseconds, and this pattern could involve all or almost all tubulin dimers. Relatively long events like visual perception, on the other hand, may take place almost continuously. As one moves upward through the spatio-temporal hierarchy, for example from tubulin to microtubules or from microtubules to neurons, a coarse-graining mechanism may play a role in filtering signal from noise (White et al., 2000), where very rapid state changes at the lower levels of the hierarchy are not perceived by the higher levels. This hierarchical filtering is consistent with the boundary-condition architecture proposed in Section 3, where the DMN’s slow fluctuations set the conditions under which faster quantum processes either maintain coherence or decohere, without needing to interact with each quantum event directly.

These energetic constraints have direct implications for the switching mechanism proposed in this paper. The analysis establishes that the brain operates under tight metabolic budgets at every level of the hierarchy, and the allocation of energy between self-evaluative processing and coherence maintenance represents a meaningful trade-off within these constraints. The sensitivity of this trade-off may be considerable, as the anesthesia research reviewed in Sections 4 and 5 demonstrates that relatively modest perturbations in energy supply and quantum infrastructure can shift the system across coherence thresholds. Winegar and MacIver (2006) found that clinical concentrations of isoflurane depressed glutamatergic EPSPs by roughly 60% while basic spike conduction persisted, which was sufficient to abolish consciousness. Kalra et al. (2023) showed that anesthetics reduced microtubule exciton diffusion lengths by only 12% to 15%, and Wiest (2025) argued that disruptions of this kind would impair the large-scale coherent quantum states in microtubules that may support conscious experience. Khan et al. (2024) then provided a causal link between microtubule integrity and consciousness, as rats given microtubule-stabilizing drugs took significantly longer to lose consciousness under isoflurane. Taken together, these findings point toward a system operating near its coherence thresholds, where relatively small changes in energy supply or quantum infrastructure can produce large-scale transitions in conscious state. Considering this, the 40% higher metabolic rate in the PCC that Carhart-Harris et al. (2014) reported represents a substantial variable within an energy budget that appears sensitive to these thresholds. The hierarchical analysis presented in this section helps to quantify what may become available for coherence maintenance when self-evaluative processing decreases, and even fractional reallocations of the neuronal energy budget could be sufficient to shift the system’s position on the quantum-classical spectrum. The fractal organization of information-processing timescales also provides context for the multi-timescale integration discussed in Section 6, as the nested hierarchy of clocking rates from tubulin (nanoseconds) through microtubules (microseconds) through neurons (milliseconds) to the whole brain (seconds) maps onto the boundary-condition architecture proposed for the DMN’s influence on quantum coherence dynamics. It is worth noting that the energy estimates in this section used Libet et al. (1979) conservative 500 ms pre-processing time as the characteristic timescale for conscious perception, and as the following section will examine, the actual operational timescales for mode switching may be considerably faster, on the order of 95 to 100 ms (Leroy and Cheron, 2020; Kurian, 2025), which would make the energy allocation trade-offs described here even more constrained.

## 6 Multi-timescale integration

### 6.1 The timescale problem

A potential objection to the proposed mechanism would involve its timing as one may wonder how can slow DMN fluctuations, which operate on timescales of seconds to minutes, affect quantum processes that operate in picoseconds. This section addresses this apparent problem by examining how the mechanism operates through a hierarchy of nested timescales.

### 6.2 The timescale hierarchy

The processes involved in the proposed mechanism span approximately fifteen orders of magnitude, from sub-picosecond quantum events to extended DMN state changes on the order of minutes. Each level in this hierarchy sets boundary conditions for the level below it.

At the fastest timescale, superradiant emission in microtubule tryptophan networks operates at approximately 0.2 picoseconds. The Fröhlich condensate dynamics described in Section 5 support these processes, and as Cavaglià et al. (2023) calculated the predicted coherent modes of dipole oscillations being in the 10^11^–10^12^ Hz frequency range translate the lifetimes of the quantum condensates to range from 10 to 1,000 ns time or possibly longer. Electromagnetic fields may propagate from the axonal membrane to microtubule bundles in the neuronal cytoskeleton within approximately one nanosecond, as dipole–dipole interactions travel at speeds on the order of 10^3^ m/s (Cavaglià et al., 2023). This layering ensures that many quantum operations complete before each environmental update, and that the electromagnetic environment is well-established before filamentary computation begins. At the microsecond scale, Singh et al. (2021c, 2021a) found that filamentary oscillations change approximately 250 microseconds before ionic firing begins, which is a pre-firing stage of computation that occurs roughly a thousand times faster than classical membrane-level spike initiation.

More complex neural firing operates on millisecond timescales and represents the classical signal propagation that neuroscience has traditionally studied. At the slowest timescales, DMN state changes and LC-NE mode shifts operate over seconds to minutes. Van der Linden et al. (2021) describe how the LC-NE system regulates decisions regarding task engagement versus disengagement and with respect to the control of DMN activity. These slow fluctuations set the boundary conditions that determine whether faster quantum processes can proceed coherently or are continuously collapsed by self-evaluative electromagnetic activity.

### 6.3 Boundary conditions and nested dynamics

The key to understanding multi-timescale integration is that slower processes do not need to directly interact with faster processes moment by moment, as they set the conditions under which faster processes operate. Carhart-Harris et al. (2014) describe the DMN as occupying the highest level of a functional hierarchy in the brain, which sets boundary conditions for processing throughout the system. From this framework’s perspective, when self-evaluative processing is high, the electromagnetic environment would contain more interactions that accelerate decoherence, and when self-evaluative processing is low, the electromagnetic environment would permit coherence to persist longer. The DMN does not need to interact with each quantum event because it shapes the environment in which those events occur.

Kurian (2025) provides a quantitative framework for understanding these relationships such that a cytoskeletal fiber of length ℓ = 1 μm and a total mass of 10^−7^ ng, with 10^4^ distinct (pointer) states, would exhibit a time accuracy of Δt = T/n ≈ mℓ^2^/nℏ = 95 ms, which approximately amounts to the average duration of a human eye blink. It also coincides with the lifetimes of the subradiant states of microtubules and other protein complexes. This indicates how quantum-level processes can integrate up to timescales relevant for conscious experience.

### 6.4 Pre-firing computation

A potential concern is how microtubule-level quantum coherence could influence classical neuronal firing without contradicting established neurophysiology. The present framework does not propose that microtubules generate action potentials or replace synaptic signaling. Instead, it aligns with evidence that cytoskeletal dynamics participate in a pre-firing stage of computation that biases spike timing and probability, both within individual neurons and across neural networks.

Singh et al. (2021c) discovered that filamentary oscillations change approximately 250 microseconds before ionic firing begins which is much before the ionic potential builds up (~500 μs). This creates a temporal window within which quantum processing could influence which neurons fire and when. They further demonstrated that the filamentary network actively directs ionic output by finding that the microfilament oscillations set the directions at which the ions are pumped and also affect their quantities. When they dissolved the axon core using depolymerizing drugs, the neuron still fired, but precise directional control was lost.

Furthermore, Singh et al. (2021a) provided experimental evidence that this filamentary network can be modulated electromagnetically as they found that when sub-threshold pulses were applied to the membrane along with MHz-range electromagnetic signals directed at the microtubule-neurofilament core, the neuron fired despite the pulse being below normal threshold. Conversely, above-threshold pulses could be silenced by applying specific frequencies that disrupted the filamentary network. The conclusion is that a neuronal membrane fires even without the cytoskeletal filaments in the interior. However, the filaments modulate the spike frequency and this modulation matters for cognition since controlling the time gaps between spikes is the aspect of the brain’s information processing.

Importantly, a hierarchical organization emerges as this filamentary circuit extends beyond individual neurons. To this end, Singh et al. (2021c) found that network’s energy is minimized when filaments of the neighboring neurons act as coupled electromagnetic oscillators, bypassing synaptic wiring, and exchanging energy. They describe the filamentary circuit as primary, with the visible geometry of neural branches being considered the neuron’s secondary circuit. In their view the filamentary circuit directs the creation of new branches, delinking of a branch and the orientation of the branches. When they dissolved the microfilament core chemically, the filamentary wiring disappeared, and all the neurons fired as if no neighbors existed. This suggests that cytoskeletal filaments are necessary for coordinated network behavior. Singh et al. (2021b) further showed that filamentary electromagnetic signaling operates approximately 1,000 times faster than ionic nerve spikes, which provides a temporal window for rapid network-wide coordination before classical neural firing occurs. The electrical properties of microtubules that underlie these pre-firing dynamics have received independent confirmation from Mohsin et al. (2025), who demonstrated through a multi-scale electrokinetic model that microtubule oscillatory behavior at gamma-band frequencies (~39 Hz) emerges from the intrinsic nanopore architecture of the polymer. Their model predicts that these oscillations persist with low damping, with soliton amplitudes requiring approximately 0.84 s to decay by 37%, which places the functional persistence of microtubule oscillatory dynamics on timescales directly relevant to cognitive processing and would allow approximately eight to nine of the integration cycles described by Kurian (2025) to complete within a single soliton’s functional lifetime. This points to microtubules operating as active dual-regime substrates, where classical ionic signal propagation and quantum coherent energy migration coexist within a single molecular architecture, with the balance between these modes potentially governed by the electromagnetic boundary conditions described in the preceding sections.

Saxena et al. (2020) discovered that this coordination operates through self-similar resonance patterns that span from individual proteins to whole neurons. Despite size variation spanning 10^6^ orders of magnitude, resonance frequency ratios remained consistent, which suggests information flows between molecular and cellular scales through resonant coupling. Interestingly they found that microtubules actively generate coherent output from incoherent input, which may imply the substrate’s default tendency is toward coherent operation. Several complementary mechanisms may support this cross-scale integration. For example, Cavaglià et al. (2023) proposed that within the neocortex, multiple pyramidal cell axons are almost parallel, and hence are capable of producing multiple cylindrical waves interfering with each other potentially resulting in electromagnetic pattern formation, which could encode information. Babcock et al. (2024) further suggested that axons may function as quantum waveguides confining superradiant optical modes to one dimension. In fact, Nishiyama et al. (2024a) calculations indicate a Fresnel number of approximately 10^−6^ for superradiant emissions supporting wide diffraction of photonic signals through neural tissue. These mechanisms are not mutually exclusive and may indeed offer parallel information processing on different size and time scales via different communication channels (Toole et al., 2018).

From this perspective, quantum coherent processing would operate upstream of classical firing, as it would shape the space of available trajectories before spike emission. When coherence persists, the microtubule network could explore possible firing trajectories in parallel, as interference dynamics would favor responses that resonate with the system’s current sensory and motor context. Then “collapse” would select among competing possibilities that would then be implemented through conventional electrophysiological mechanisms, and from the present framework’s perspective, this pre-firing computation occurs in conditions determined by the current DMN state. When self-evaluative activity is low, the 250-microsecond window permits more extensive quantum exploration before classical firing patterns are determined. When self-evaluative activity is high, decoherence occurs faster, and pre-firing computation proceeds more classically, as it defaults to sequential manipulation of stabilized, stored states that are characteristic of System 2. Kurian (2025) calculation that cytoskeletal fibers exhibit time accuracy of approximately 95 milliseconds provides a quantitative basis for how quantum-level results integrate up to timescales relevant for conscious experience.

## 7 Cognitive-level consequences

The mechanism proposed in Sections 3 through 5 naturally leads to predictions about cognitive phenomena that should accompany shifts between quantum and classical processing modes. If self-evaluative DMN activity modulates microtubule quantum coherence, then cognitive states characterized by reduced self-monitoring should exhibit different cognitive signatures than states with high self-evaluation.

### 7.1 Flow states and transient Hypofrontality

Leroy and Cheron (2020) recorded EEG dynamics during tightrope performance and compared flow states to stress states. Their results support the assertion that flow emergence requires transient hypofrontality. During flow states, they observed decreased activation in BA10, which is a region involved in metacognition, with simultaneous increased activity in basal ganglia structures involved in motor coordination. The stress state showed the opposite pattern. During flow, an alpha peak was present which disappeared during stress. The temporal dynamics showed during the first 100 ms after TA activation event-related synchronization (ERS) and was present during the flow while stress states showed desynchronization. This 100-millisecond switching capability demonstrates the brain can transition between modes very quickly.

The regional specificity of alpha oscillations connects to an apparent puzzle as Carhart-Harris et al. (2014) found that alpha power in the posterior cingulate cortex explained 66% of the variance in ego dissolution experiences as alpha decreases correlated with participants’ reported reduced sense of self. At first glance, this seems to conflict with how alpha is present during flow. But the resolution between these lies in where the alpha originates in the brain as Carhart-Harris et al. measured alpha in the PCC, which is a DMN hub for self-referential processing, while Leroy and Cheron found the flow-related alpha in basal ganglia structures that are involved in the automaticity of motor function. From the energy economy perspective developed in Section 5, when metabolic energy is not directed to self-referential processing in DMN regions, it may become available for task-relevant functions. This would explain why the alpha signature in Leroy and Cheron’s experiments shifts from DMN regions to basal ganglia regions during flow, while the psychedelic ego dissolution that Carhart-Harris et al. observed involves DMN suppression without the comparable compensatory task-related activation.

These cognitive state differences may have field-level and quantum-level correlates as MacIver (2022) used chaotic attractor analysis of EEG signals, and he found that different conscious states have their own distinct geometries. In this, waking consciousness shows the shape of spherical attractor clouds, but when affected by anesthesia, they collapse into flattened ellipsoids. This geometric shift may reflect the transition from a high-dimensional coherent regime to a lower-dimensional classical one.

At the quantum level, Kerskens and López Pérez (2022) detected zero-quantum-coherence signals in living human brains that had no correlates with any classical NMR contrast mechanism. These signals depended on conscious awareness, which declined when participants fell asleep, and their complexity correlated with short-term memory performance in related work (López Pérez et al., 2023). These findings suggest entanglement mediated by consciousness-related brain functions. While their methodology detects nuclear spin correlations instead of the electronic coherence proposed in the present framework, the finding that non-classical correlations in the brain track conscious state is consistent with the broader proposal that quantum processes play a functional role in cognition.

### 7.2 Insight, flow, and the gamma burst

Kounios and Beeman (2014) define insight as “any sudden comprehension, realization, or problem solution that involves a reorganization of the elements of a person’s mental representation of a stimulus, situation, or event to yield a nonobvious or nondominant interpretation.” According to their research, awareness of such representational change, while abrupt, takes place after a period of unconscious processing. Moreover, because insights are generally a product of unconscious processing, their emergence appears to be disconnected from the ongoing conscious thought processes. Conversely, analytic thought is deliberate and conscious and is characterized by incremental approach to finding a solution. This characterization maps onto the System 1 and System 2 distinction, where insight emerges from parallel unconscious processing while analytic thought proceeds through sequential conscious steps. Vervaeke et al. (2018) extend these ideas by characterizing flow as “an extended ‘aha!” and conclude that flow represents neither a deliberative nor an automatic form of processing, but instead represents a separate category of “spontaneous thought.”

In earlier work, Kounios and Beeman (2009) found that preparation for solving an upcoming problem with insight involved directing attention inwardly while analytical solving involved directing attention outwardly. The inward orientation involves letting unconscious associative networks find solutions without self-monitoring and having the ability to notice when these insights arise. Insight also requires a temporary reduction in interfering visual inputs through alpha waves. This pattern resembles the common experience of insights arriving during low-demand activities like showering or driving, where active self-monitoring is reduced. Most significantly, they found that insight solutions were associated with a burst of high-frequency (i.e., 40-Hertz gamma-band) activity starting about 300 milliseconds before the button-press representing a discrete transition from a state of no conscious information about the solution to the final complete solution, with no intermediate states (Kounios and Beeman, 2014).

From the perspective of this paper’s mechanism, these findings converge as sensory gating and reduced self-referential attention would create conditions for extended quantum coherence, unconscious associative processing would occur in the coherent regime, and the gamma burst would mark the collapse event that brings the solution into classical, reportable awareness. The discrete, all-or-nothing character of insight, with no intermediate states, aligns with what collapse dynamics would predict, and flow would represent a sustained version of this same regime.

## 8 Discussion

### 8.1 The current state of the debate

The evidence reviewed in this paper supports the proposal that quantum coherence may provide the computational substrate for System 1 processing, and self-evaluative DMN activity controls the gradient of coherence-to-decoherence that determines the brain’s position on the quantum-classical spectrum. This hypothesis aligns with the contemporary dual process position articulated by Evans and Stanovich (2013), who reframed System 1 and System 2 terminology as Type 1 and Type 2 processing because they wanted to avoid implying that these may be discrete anatomical systems without mechanistic justification. In their account, rapid autonomous processes yield default responses, and higher order reasoning intervenes when required. In this, Type 2 heavily relies on working memory and supports hypothetical reasoning. The present framework suggests that middle ground can be found between Kahneman (2011) original descriptions and Evans and Stanovich (2013) interpretation by treating System 1 and System 2 as distinct operating regimes of a single biological substrate. For the purposes of this paper, System 1 refers to processing that preserves indeterminate internal states long enough to support parallel evaluation across multiple possibilities before commitment, and System 2 refers to processing that commits early to discrete, definite states that are then manipulated serially in working memory. From this perspective, the distinction reflects a shift in the timing of commitment within a single substrate instead of there being a literal on and off switch between two separate systems.

### 8.2 Relationship to predictive processing

Carhart-Harris and Friston (2019) characterize the brain as a hierarchical prediction machine where high-level priors constrain lower-level processing, and their REBUS model proposes that psychedelics relax these priors to permit greater cognitive flexibility. The present framework offers a physical mechanism for these dynamics of predictive processing. Within the REBUS model, beliefs are maintained through what Carhart-Harris and Friston call precision-weighting on prior expectations. In their model, precision is statistically equivalent to inverse variance (i.e., negative entropy). This statistical relationship has direct implications for the present framework, as high precision corresponds to low entropy and therefore to a narrow, definite belief state, while low precision corresponds to high entropy and therefore to an uncertain state spread across multiple possibilities. This connects predictive processing to the quantum cognition findings reviewed in Section 1 (Kvam and Pleskac, 2017; Busemeyer et al., 2019, 2020).

Carhart-Harris and Friston (2019) describe how precision-weighting shapes the geometry of neural dynamics, so that a loss of precision (confidence) in posterior beliefs corresponds to a decrease in the curvature of the free-energy landscape associated with the neuronal activity encoding those beliefs. When precision is high, the landscape has steep curvature that constrains processing to narrow attractor basins, and when the system’s precision relaxes, the landscape flattens which allows for a broader exploration. They further characterize the DMN as the brain’s “Centre of Gravity”. This characterization positions the DMN as the natural locus for a global coherence-modulating mechanism, which aligns with the present framework’s proposal.

### 8.3 Testable predictions

The proposed mechanism generates predictions that can be tested with current and near-future experimental methods.

#### 8.3.1 Qualitative predictions

First, specific electromagnetic signatures should correlate with the mode of processing. Flow states and insight should show reduced electromagnetic complexity in DMN regions, while analytical processing and states of anxiety should show increased electromagnetic complexity in these regions. MacIver (2022) chaotic attractor analysis provides a potential methodology, based on the qualitative observations where 3-D clouds generated for subjects who were awake and conscious were largely spherical, reflecting many degrees of freedom and complex brain activity while the loss of consciousness (LOC) produced 3-D clouds that collapsed into ellipsoid shapes. Therefore, flow states might show a reorganization of attractor geometry in the EEG and MEG data, as complexity may be preserved but redistributed away from self-referential regions.

Second, interventions that modulate DMN activity should shift the quantum-classical balance. Meditation practices that reduce DMN activation should extend coherence windows and enhance System 1 processing. Anxiogenic interventions that increase DMN activation should accelerate decoherence and shift processing toward System 2. Carhart-Harris et al. (2014) provide support for this prediction, as they found decreased DMN-TPN anticorrelation in experienced meditators during rest and noted that decreased DMN-TPN inverse coupling is especially marked during meditation referred to as ‘non-dual awareness’.

Third, microtubule-binding compounds should affect consciousness onset and offset predictably. Khan et al. (2024) already demonstrated this with a large effect size (Cohen’s *d* = 1.9), as rats given brain-penetrant microtubule-binding drugs took significantly longer to fall unconscious under isoflurane. The framework predicts that microtubule-stabilizing compounds should delay consciousness loss under any anesthetic that acts primarily through microtubule disruption.

Fourth, flow state induction should show reduced markers of microtubule decoherence. If the Kerskens and López Pérez (2022) zero-quantum-coherence signals can be reliably measured, flow states should show enhanced or differently organized coherence signatures compared to stressed or self-monitoring states.

#### 8.3.2 Quantitative predictions

The energy threshold analysis from Section 5 generates specific quantitative predictions as Cavaglià et al. (2023) calculate that coherence maintenance requires energy pumping above 0.3 k_B_T/s, while action potentials supply 5 to 50 k_B_T/s under normal waking conditions.

The framework predicts that unconsciousness should occur when action potential rate falls below a certain threshold. There should be signs of significant loss of coherence in MTs at this threshold. This prediction can be tested against detailed measurements of action potential rates during anesthesia induction.

The framework also predicts that quantum-dominant processing (flow, insight) and classical-dominant processing (analytical, anxious) should show different metabolic energy distributions across brain regions, and these differences should not be reducible to simple activity level differences. PET or metabolic MRI studies comparing these states should reveal redistribution of energy away from DMN regions during quantum-dominant states.

Finally, under conditions of energy scarcity (fasting, dehydration, exhaustion), the framework predicts that an energy trade-off should emerge, but the nature of this trade-off may depend on the task at hand and the individual’s training or disposition. One possibility is that processing that requires extended coherence degrades first because coherence maintenance is energetically expensive. Another possibility is that self-evaluative processing gets deprioritized first considering the DMN’s vast energy usage (Carhart-Harris et al., 2014), which would both conserve the energy normally spent on DMN activity and permit extended coherence for task-relevant quantum processing. Runners’ high and similar phenomena during extreme exertion may reflect this second strategy, where the body prioritizes energy for movement over self-monitoring, and the suppression of self-evaluation produces the characteristic altered state. Which system gets deprioritized first, and under what conditions, remains an open question that could be investigated by examining System 1 versus System 2 performance across different task demands and individual differences in contemplative, athletic training, music performance, or parallel computation (see Table 2).

**Table 2:** Summary of testable predictions generated by the framework.

Prediction	Measurable variable	Experimental approach
Flow states and insight show reduced electromagnetic complexity in DMN regions	EEG/MEG power distributions and attractor geometry in PCC and BA10	Within-subject comparison of flow and anxiety conditions using MacIver (2022) chaotic attractor analysis
DMN-suppressing interventions extend coherence windows and enhance system 1 processing	DMN-TPN anticorrelation strength and system 1 task performance	Pre-post meditation or anxiogenic intervention with concurrent fMRI or EEG
Microtubule-stabilizing compounds delay anesthetic-induced unconsciousness	Time to loss of consciousness under titrated anesthesia	Extension of Khan et al. (2024) protocol to additional anesthetic agents and microtubule-binding compounds
Flow states show enhanced non-classical neural correlations	Zero-quantum-coherence NMR signals	Flow-state induction with concurrent NMR measurement comparing flow, stress, and rest conditions (Kerskens and López Pérez, 2022)
Unconsciousness onset correlates with action potential rate falling below coherence threshold	Cortical action potential firing rate during anesthesia induction	Graded anesthetic dose–response with electrophysiological recording, compared to Cavaglià et al. (2023) 0.3 kBT/s minimum
Quantum-dominant and classical-dominant processing show different metabolic energy distributions	Regional cerebral metabolic rate of glucose or oxygen	PET or metabolic fMRI comparing flow, analytical, and resting conditions
Energy scarcity produces differential degradation of system 1 vs. system 2 depending on task demands and training	System 1 vs. system 2 task performance under metabolic stress	Cognitive testing battery under fasting or exhaustion conditions with individual differences in contemplative or athletic training as moderating variables

### 8.4 Limitations

A few limitations should be acknowledged. First, while the individual components of the proposed causal chain draw on empirical findings, the strength of evidence varies across links, with some connections supported by direct experimental measurement and others remaining physically motivated but not yet directly demonstrated in the specific configuration proposed here. The complete chain has not been tested as an integrated mechanism. In particular, the quantitative relationship between variations in self-evaluative electromagnetic activity and changes in microtubule decoherence rates has not been directly characterized in living neural tissues, and establishing the physiological sufficiency of this link remains among the most important empirical goals for testing the framework. Second, the quantitative predictions depend on parameters that are not yet precisely measured in living neural tissue. The energy thresholds, coherence timescales, and decoherence rates discussed in this paper derive largely from *in vitro* measurements and theoretical calculations, and *in vivo* confirmation remains an important goal. This challenge is compounded by the fact that most neuroimaging techniques such as EEG and fMRI capture signals at spatial and temporal scales which are far removed from the quantum processes proposed to underlie consciousness. MacIver (2022) notes that the electromagnetic patterns most relevant to consciousness may be those propagating toward the brain’s central regions and interacting with intracellular structures, which would be largely invisible to scalp-based recordings. The work of Kerskens and López Pérez (2022) represents one attempt to bridge this measurement gap through zero-quantum-coherence signals, and replication and extension of an approach like this may be valuable for establishing whether similar signals correlate with the processing mode shifts proposed here.

## 9 Conclusion

Busemeyer et al. (2019, 2020) demonstrated that human cognition follows quantum probability models under conditions of low confidence and classical Markov models under conditions of high confidence. They described this as decoherence through interaction with a noisy mental environment, but they did not identify the biological source of this noise or the mechanism that would control the transition. The present paper proposes that self-evaluative DMN processing may generate the electromagnetic environment that modulates microtubule quantum coherence, and that this modulation may constitute the biological switch between quantum and classical processing modes. From this perspective, the self-evaluative electromagnetic activity proposed here may constitute the biological source of the “noisy mental environment” that Busemeyer et al. (2019, 2020) identified as driving quantum-to-classical transitions in their cognitive models but did not specify.

The evidence supporting this proposal spans multiple levels of analysis. At the cellular level, microtubule tryptophan networks exhibit quantum energy migration, superradiant enhancement, and thermal robustness that are consistent with coherent processing at physiological temperatures (Kalra et al., 2023; Babcock et al., 2024; Patwa et al., 2024). At the neural level, electromagnetic fields have been shown to propagate to intracellular structures including the cytoskeleton via ephaptic coupling, electrodiffusion, and mechanotransduction (Pinotsis et al., 2023), and calcium signaling can transduce neural activity patterns to the microtubule electrostatic environment (Wiest, 2025). At the cognitive level, flow states require transient hypofrontality and DMN suppression (Leroy and Cheron, 2020; Menon, 2023), insight requires reduced self-monitoring and sensory gating (Kounios and Beeman, 2009), and the level of confidence predicts whether quantum or classical models better fit decision-making (Busemeyer et al., 2019, 2020). At the pharmacological level, anesthetics reduce microtubule quantum efficiency (Kalra et al., 2023), protecting microtubules delays unconsciousness (Khan et al., 2024), and the Meyer-Overton correlation is best explained by microtubule binding (Craddock et al., 2017; Wiest, 2025).

The framework embeds quantum processes in neurophysiology by connecting them to the locus coeruleus-norepinephrine system, the Default Mode Network, membrane dipole dynamics, calcium signaling, and metabolic energy allocation. By identifying the DMN as the control mechanism and self-evaluation as the cognitive trigger, the framework connects quantum processing to everyday psychological phenomena like flow, confidence, and insight. While the complete causal chain remains to be tested as an integrated mechanism, the framework generates specific predictions assessable with current neuroimaging methods, pharmacological interventions, and behavioral studies.

Dual-process theory has provided a valuable descriptive framework for conceptually understanding the modes of processing, but it has lacked a mechanistic explanation for how these modes operate and how the brain switches between them. The present paper proposes that the answer lies in the brain’s ability to control its own position on the quantum-classical spectrum, with self-evaluative processing functioning as the switch that determines to what degree quantum coherence in microtubules is maintained.

## Data Availability

The original contributions presented in the study are included in the article/supplementary material, further inquiries can be directed to the corresponding author.
